# ﻿Complete mitochondrial genome of *Lepidocephalichthysberdmorei* and its phylogenetic status within the family Cobitidae (Cypriniformes)

**DOI:** 10.3897/zookeys.1221.129136

**Published:** 2024-12-10

**Authors:** Min Zhou, Cheng Wang, Ziyue Xu, Zhicun Peng, Yang He, Ying Wang

**Affiliations:** 1 Hubei Engineering Research Center for Protection and Utilization of Special Biological Resources in the Hanjiang River Basin, Jianghan University, Wuhan, China Jianghan University Wuhan China; 2 State Key Laboratory of Freshwater Ecology and Biotechnology, Institute of Hydrobiology, Chinese Academy of Sciences, Wuhan, China Institute of Hydrobiology, Chinese Academy of Sciences Wuhan China; 3 Academy of Plateau Science and Sustainability, Qinghai Normal University, Xining, China Qinghai Normal University Xining China

**Keywords:** Gene arrangement pattern, *Lepidocephalichthysberdmorei*, mitochondrial genome, phylogenetic analysis

## Abstract

In this study, the complete mitochondrial genome of *Lepidocephalichthysberdmorei* was first determined by the primer walking sequence method. The complete mitochondrial genome was 16,574 bp in length, including 13 protein-coding genes (PCGs), 22 transfer RNA (tRNA) genes, two ribosomal RNA (rRNA) genes, and a control region (D-loop). The gene arrangement pattern was identical to that of other teleosts. The overall base composition was 29.9% A, 28.5% T, 25.5% C, and 16.1% G, with an A+T bias of 58.4%. Furthermore, phylogenetic analyses were conducted based on 13 PCGs from the mitochondrial genomes of 18 cobitid species using with three different methods (Neighbor-joining, Maximum likelihood, and Bayesian inference). All methods consistently showed that the four species of the genus *Lepidocephalichthys* form a monophyletic group. This study would provide effective molecular information for the *Lepidocephalichthys* species as well as novel genetic marker for the study of species identification.

## ﻿Introduction

*Lepidocephalichthysberdmorei* (Blyth 1860)belongs to the genus *Lepidocephalichthys* within the family Cobitidae, which is widely distributed in the Irrawaddy, Sittang, Salween, Chao Phraya, Mekong basins of Burma, Thailand, and China (Kottelat and Lim 1993). According to FishBase, there are approximately 25 valid species in the genus *Lepidocephalichthys* (Froese and Pauly 2024). The lack of reliable morphological characteristics, coupled with the widespread misapplication of names, has made it challenging to differentiate this species from its close relatives. For instance, the close resemblance in physical features between *L.thermalis* and *L.berdmorei* poses a significant challenge in morphological differentiation (Kottelat 2012). Therefore, molecular information is necessary for an additional method to delimit and identify species. *Lepidocephalichthysberdmorei* is a small-sized freshwater fish species, that inhabits hill swift streams, and lakes with sandy and gravel bottoms (Kamei et al. 2023). In recent years, due to over-exploitation, damage to spawning beds, and construction of the hydroelectric dam in the Lancang River, the wild population size of *L.berdmorei* has declined dramatically (Buj et al. 2015; Zhang et al. 2019).

The mitochondrial genome (mtDNA) is a circular double-stranded molecule consisting of 13 PCGs, 22 tRNAs, two rRNAs, and a control region (D-loop) (Anderson et al. 1981; Boore 1999; Shen et al. 2020; Chu et al. 2022; Jia et al. 2023). Traditional morphological and biological approaches have focused on the ecological characteristics of populations and reproduction, with relatively little molecular research in the genus *Lepidocephalichthys* (Gohain and Deka 2017; Trif et al. 2022). Because of its limited recombination, highly conserved gene content, maternal inheritance and moderate evolutionary speed, mtDNA is now widely used to study population genetics, phylogeny, and species identification (Avise et al. 1987; Harrison 1989; Boore and Brown 1998; Ballard and Whitlock 2004; Galtier et al. 2009; Sureandiran et al. 2023). As proof, Wang et al. (2021) successfully identified fish species from the Xiangjiaba reservoir in Jinsha River using mitochondrial DNA barcoding. Goswami et al. (2022) characterized the genetic diversity of ten loaches from northeastern India based on sequence fragments of *cox1*, *cytb*, and *16S rRNA* genes; Zhang et al. (2023) demonstrated that the evolutionary position of *Rectorisluxiensis* (Wu et al. 1977) was consistent with traditional taxonomy through phylogenetic analysis of mitochondrial genomes. Currently, four mitochondrial genomes have been reported in NCBI databases, including *L.micropogon* (Blyth 1860), *L.guntea* (Hamilton 1822), *L.hasselti* (Valenciennes and Cuvier 1846), and *L.annandalei* (Chaudhuri 1912). Nevertheless, the complete mitochondrial genome of *L.berdmorei* has not been reported until now.

In this study, the complete mitochondrial genome of *L.berdmorei* was sequenced for the first time. The variation in tRNA length, position, and size of the control region, and the codon usage bias were analyzed. Subsequently, the 13 PCGs were concatenated and utilized, with those of other cobitids, to confirm the phylogenetic position of *L.berdmorei*. Therefore, these findings will provide valuable information and contribute to future species comparison and evolutionary research.

## ﻿Materials and methods

### ﻿Sample collection and DNA extraction

An adult individual of *L.berdmorei* was obtained in 2020 from the Mengla town, Xishuangbanna Dai Autonomous Prefecture, Yunnan Province, China (21°57'70"N, 101°60'54"E) (Suppl. material 1: fig. S1). Species were identified using the original morphological descriptions in the Fauna Sinica field guides (Chen 1998). After initial morphological identification, the specimen was deposited in the Animal Genetics Center of Jianghan University under the voucher number JHU202012029. A 40–50-mg fin clip was collected and preserved in 95% ethanol at 4 °C. Total genomic DNA was extracted from caudal fin tissue using the traditional phenol-chloroform method (Sambrook and Russell 2001).

### ﻿Mitogenome sequencing, assembly, and annotation

Eight pairs of primers (Suppl. material 1: table S1) were designed based on the mtDNA sequences of closely allied species. The PCR conditions were as follows: initial denaturation at 94 °C for 2 min, then 35 cycles of denaturation at 94 °C for 30 s, annealing at 55 °C for 30 s, and extension at 72 °C for 1 min, followed by the final extension at 72 °C for 10 min. All obtained fragments were quality-proofed and searched via BLAST in the NCBI database to confirm that the amplicon is the actual target sequence.

Sequences were assembled manually by the Seqman program using DNAstar v. 7.1 software (Burland 2000). The mitochondrial genome was annotated roughly following the procedure described before (Wang et al. 2011, 2018). The PCGs, rRNA genes, tRNA genes, and one control region of the mitochondrial genome were annotated by MitoAnnotator (http://mitofish.aori.u-tokyo.ac.jp/annotation/input.html) (Iwasaki et al. 2013). Their secondary structures of tRNAs were predicted by tRNAScan-SE (http://lowelab.ucsc.edu/tRNAscan-SE/; Lowe and Eddy 1997) and Forna (force-directed RNA) (Kerpedjiev et al. 2015).

The base composition and relative synonymous codon usage (RSCU) of the mitogenome were calculated and produced using PhyloSuite v. 1.2.3 (Zhang et al. 2020) and MAGA X (Kumar et al. 2018). The formulas to calculate the nucleotide composition of skew are as follows: AT-skew = (A – T)/ (A + T) and GC-skew = (G – C)/ (G + C) (Perna and Kocher 1995).

### ﻿Phylogenetic analyses

To verify the phylogenetic position of *L.berdmorei*, 17 mitogenome sequences from GenBank were retrieved (Suppl. material 1: table S2; Saitoh et al. 2006, 2010). The 13 PCGs for each species were concatenated and then aligned by program MAFFT using default settings (Katoh et al. 2002), and phylogenetic analyses were performed using Neighbor-joining (NJ), Maximum likelihood (ML), and Bayesian inference (BI) methods. To root the phylogenetic tree, *Syncrossusbeauforti* (Smith 1931)and *S.hymenophysa* (Bleeker 1852) from Botiidae were chosen as outgroups.

A NJ phylogenetic tree was constructed using MEGA 7 (Kumar et al. 2016) with 1,000 bootstrap replicates. The ML method was assembled in RAxML 7.0.3 (Stamatakis 2006), with 1,000 bootstrap replicates. GTR + F + I + G4 was selected as best-fit model according to Bayesian Information Criterions (BIC) estimated by ModelFinder (Kalyaanamoorthy et al. 2017). The BI phylogeny was carried out using MrBayes v. 3.2.7a (Ronquist et al. 2012) under the best-fit models with 5,000,000 generations in two runs of eight chains each.

#### ﻿Abbreviations

Mitogenome, mitochondrial genome; **mtDNA**, mitochondrial DNA; **PCGs**, protein-coding genes; **tRNA**, transfer RNA; **rRNA**, ribosomal RNA; ***atp6*** and ***atp8***, ATPase 6 and ATPase 8; **cox1–3**, cytochrome oxydasec subunits I–III; ***cytb***, cytochrome b; **LA-PCR**, long and accurate polymerase chain reaction; ***nd1–6***, NADH dehydrogenase subunits 1–6; ***nd4l***, NADH dehydrogenase subunits 4L; **A+T**, A+T rich region; **RSCU**, relative synonymous codon usage; **trnA**, *tRNA^Ala^*; **trnC**, *tRNA^Cys^*; **trnD**, *tRNA^Asp^*; **trnE**, *tRNA^Glu^*; **trnF**, *tRNA^Phe^*; **rrnS**, *12S rRNA*; **rrnL**, *16S rRNA*; **trnG**, *tRNA^Gly^*; **trnH**, *tRNA^His^*; **trnI**, *tRNA^Ile^*; **trnK**, *tRNA^Lys^*; **trnL1**, *tRNA^Leu^*^(*TAA*)^; **trnL2**, *tRNA^Leu^*^(*TAG*)^; **trnM**, *tRNA^Met^*; **trnN**, *tRNA^Asn^*; **trnP**, *tRNA^Pro^*; **trnQ**, *tRNA^Gln^*; **trnR**, *tRNA^Arg^*; **trnS1**, *tRNA^Ser^*^(*TGA*)^; **trnS2**, *tRNA^Ser^*^(*GCT*)^; **trnT**, *tRNA^Thr^*; **trnV**, *tRNA^Val^*; **trnW**, *tRNA^Trp^*; **trnY**, *tRNA^Tyr^*; **DHU**, Dihydrouracil; **NJ**, Neighbor-joining; **ML**, Maximum likelihood; **BI**, Bayesian inference.

## ﻿Results and discussion

### ﻿Mitogenome organization and nucleotide composition

**Figure 1.**
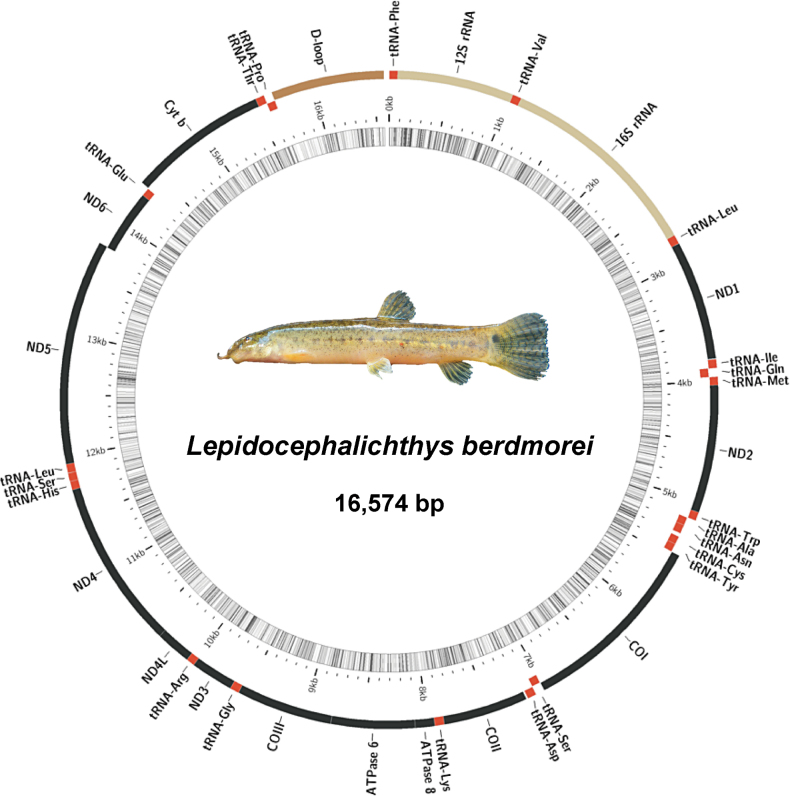
Gene map and organization of the mitochondrial genome of *Lepidocephalichthysberdmorei*. Photograph of *L.berdmorei* from https://fishbase.se/summary/Lepidocephalichthys-berdmorei.html.

The length of the complete mitochondrial genome of *L.berdmorei* is 16,574 bp (GenBank accession number: OP651767). The complete mitochondrial genome of *L.berdmorei* shares high similarity in gene arrangement, base composition, and codon usage pattern with those of other teleosts, indicating that the mitochondrial genome is highly conserved in evolution (Boore 1999; Taanman 1999; Broughton et al. 2001; Zou et al. 2019; Shen et al. 2020; Wang et al. 2020; Yu et al. 2021). The mitogenome is a circular double-stranded molecule with a highly conserved structure, consisting of 13 PCGs, 22 tRNA genes, two rRNA genes, and a control region (D-loop) (Fig. 1, Table 1).

**Table 1.** Organization of the mitochondrial genome of *Lepidocephalichthysberdmorei*.

Locus	Position		Size (bp)	Intergenic nucleotides^a^	Codon		Anti-codon	Strand^b^
	From	To			Start	Stop		
*tRNA^Phe^* (*S*)	1	69	69	0	–	–	GAA	H
*12S rRN*A	70	1019	950	0	–	–	–	H
*tRNA^Val^* (*V*)	1020	1091	72	0	–	–	TAC	H
*16S rRNA*	1092	2767	1676	0	–	–	–	H
*tRNA^Leu^*^(^*^TAA^*^)^(*L1*)	2768	2842	75	1	–	–	TAA	H
*nd1*	2844	3818	975	5	ATG	TAA	–	H
*tRNA^AIle^* (*I*)	3824	3895	72	-2	–	–	GAT	H
*tRNA^Gln^* (*Q*)	3894	3964	71	1	–	–	TTG	L
*tRNA^Met^* (*M*)	3966	4034	69	0	–	–	CAT	H
*nd2*	4035	5081	1047	-2	ATG	TAG	–	H
*tRNA^Trp^* (*W*)	5080	5148	69	2	–	–	TCA	H
*tRNA^Ala^* (*A*)	5151	5219	69	1	–	–	TGC	L
*tRNA^Asn^* (*N*)	5221	5293	73	30	–	–	GTT	L
*tRNA^Cys^* (*C*)	5324	5390	67	0	–	–	GCA	L
*tRNA^Tyr^* (*Y*)	5391	5459	69	1	–	–	GTA	L
*cox1*	5461	7011	1551	2	GTG	TAA	–	H
*tRNA^Ser^*^(^*^TGA^*^)^(*S1*)	7014	7084	71	1	–	–	TGA	L
*tRNA^Asp^* (*D*)	7086	7158	73	13	–	–	GTC	H
*cox2*	7172	7862	691	0	ATG	T	–	H
*tRNA^Lys^* (*K*)	7863	7938	76	1	–	–	TTT	H
*atp8*	7940	8107	168	-10	ATG	TAA	–	H
*atp6*	8098	8781	684	-1	ATG	TAA	–	H
*cox3*	8781	9566	786	-1	ATG	TAA	–	H
*tRNA^Gly^* (*G*)	9566	9638	73	0	–	–	TCC	H
*nd3*	9639	9989	351	-2	ATG	TAG	–	H
*tRNA^Arg^* (*R*)	9988	10056	69	0	–	–	TCG	H
*nd4l*	10057	10353	297	-7	ATG	TAA	–	H
*nd4*	10347	11729	1383	-1	ATG	TAG	–	H
*tRNA^His^* (*H*)	11729	11797	69	0	–	–	GTG	H
*tRNA^Ser^*^(^*^GCT^*)(*S2*)	11798	11866	69	1	–	–	GCT	H
*tRNA^Leu^*^(^*^TAG^*^)^(*L2*)	11868	11940	73	0	–	–	TAG	H
*nd5*	11941	13779	1839	-4	ATG	TAA	–	H
*nd6*	13776	14297	522	0	ATG	TAA	–	L
*tRNA^Glu^* (*E*)	14298	14366	69	5	–	–	TTC	L
*cytb*	14372	15512	1141	0	ATG	T	–	H
*tRNA^Thr^* (*T*)	15513	15584	72	-2	–	–	TGT	H
*tRNA^Pro^* (*P*)	15583	15652	70	0	–	–	TGG	L
D-loop	15653	16574	922	0	–	–	–	H

^a^ Negative value indicates the overlapping sequences between adjacent genes. ^b^ H: heavy strand; L: light strand.

The overall base composition is 29.9% for A, 16.1% for G, 25.5% for C, and 28.5% for T, which is consistent with the lowest frequency for G among the four bases in fish mitochondrial genomes, and revealing the A+T-rich content (58.4%) (Mayfield and McKenna 1978; Meyer 1993). Based on the analysis of nucleotide composition, this complete sequence exhibits a clear bias towards A and T (AT-skew = 0.02, GC-skew = -0.23) (Suppl. material 1: table S3). Both *L.berdmorei* and 58 species of Cobitidae exhibit an AT bias in their mitogenomes, but the A+T-rich content size varied among species, and it may be related to factors such as natural mutations and selection pressures during replication and transcription (Zhong et al. 2002; Yu et al. 2021). Hence, during the processes of replication and transcription, the asymmetry in nucleotide composition was used to infer the direction of gene orientation and replication (Francino and Ochman 1997; Frank and Lobry 1999; Satoh et al. 2016; Moeckel et al. 2023).

### ﻿Overlaps and non-coding intergenic spacers

Cobitidae mitogenomes range from 16,574 bp (*L.berdmorei*) to 16,646 bp (*Cobitisstriata* (Ikeda, 1936)) in length (Suppl. material 1: table S2). With a few exceptions, the gene arrangements of fish mitogenomes are usually conserved (Anderson et al. 1981; Chang et al. 1994; Satoh et al. 2016; Chu et al. 2022). A typical feature in the mitochondrial genome of teleosts is the overlap of nucleotides between adjacent genes, suggesting that the size of mitochondrial DNA is very compact and economical, with potential kinetic advantages during the process of replication (Boore 1999; Curole and Kocher 1999; Taanman 1999; Wang et al. 2011; Satoh et al. 2016; Zou et al. 2017; Zou et al. 2018; Zhang et al. 2023). Similarly, in the *L.berdmorei* mitochondrial genome, there are overlaps and intervals of different lengths in all genes except for trnF/rrnS, rrnS /trnV, trnV/rrnL, rrnL/trnL2, trnM/*nd2*, trnC/trnY, *cox2*/trnK, trnG/*nd3*, trnR/*nd4l*, trnH/trnS2, trnL2/*nd5*, *nd6*/trnE, and *cytb*/trnT. They have the longest spacer in trnN/trnC (30 bp) and the largest genetic overlap in *atp8*/*atp6* (10 bp) (Table 1). The length of the mitochondrial genome is related to the various overlaps and intergenic spacers between adjacent genes (Huang and Liu 2010). Interestingly, the presence of a specific 3 bp insertion (GCA) in the overlapping *atp8*–*atp6* motif of both *L.berdmorei* and other loaches compared to the conserved motif of 7 bp (ATGATAA) in other Cypriniformes fishes, suggests that this insertion is characteristic of loaches (Kanu et al. 2016; Wu et al. 2016; Yu et al. 2016; Yu et al. 2021). They may influence the expression of neighboring genes, regulate the normal operation of mitochondrial function, and participate in the process of mitochondrial genome replication and transmission (Boore 1999; Taanman 1999; D’Souza and Minczuk 2018).

### ﻿PCGs and codon usage

The length of PCGs was 11,413 bp (68.86%) and it blanketed 7 NADH dehydrogenases (*nd1*–*6* and *nd4l*), three cytochrome coxidases (*cox1*–*3*), two ATPases (*atp6* and *atp8*) and one cytochrome b (*cytb*). The size of PCGs ranged from *nd4l* (297 bp) to *nd5* (1839 bp). As in other vertebrates, the *nd6* and eight tRNA genes (*tRNA^Gln^*, *tRNA^Ala^*, *tRNA^Asn^*, *tRNA^Cys^*, *tRNA^Tyr^*, *tRNA^Ser^*, *tRNA^Pro^*, and *tRNA^Glu^*) are encoded on the light strand, and the others are encoded on the heavy strand (Fig. 1, Table 1) (Wen et al. 2017; Zou et al. 2017; Yu et al. 2021). In addition, the bias of nucleotide composition was estimated (Suppl. material 1: table S3). All 13 PCGs showed a significant negative GC-skew. It may be that mutations in the replication process or adaptive evolution cause GC-skew. However, how to explain this unusual GC-skew needs further study.

**Figure 2.**
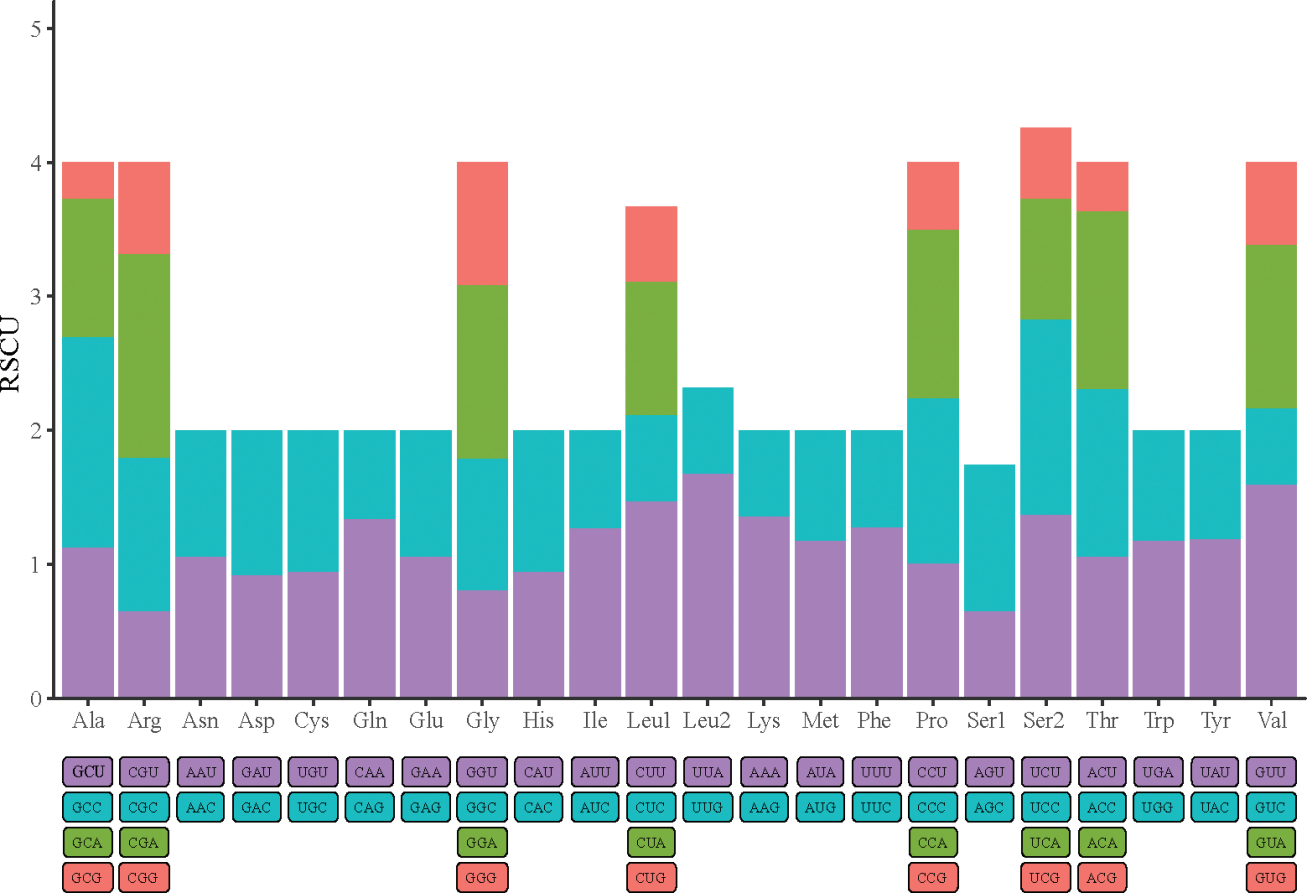
The relative synonymous codon usage (RSCU) of *L.berdmorei* mitogenome.

Further analysis revealed that among 13 PCGs, most mitochondrial genes of *L.berdmorei* started with codon ATG, while only the *cox1* gene began with codon GTG. Unconventional start codons are a common phenomenon within the mitogenomes of fish (Zhang and Shen 2019; Yu et al. 2021). Eight of the PCGs are ended by TAA termination codons. The *nd2*, *nd3*, and *nd4* genes ended with TAG stop codons. The *cox2* and *cytb* use incomplete stop codons (T-) (Table 1). The relative synonymous codon usage (RSCU) denotes the differential usage of synonymous codons encoding the same amino acid. Essentially, the RSCU value was calculated by dividing the amino acids encoded by the same codons and their probability of appearing in the same codons (Sharp and Li 1986). The RSCUs of *L.berdmorei* mitogenome (Fig. 2, Table 2) show a clear preference for the usage of A and T. The total number of codons in the *L.berdmorei* mitochondrial genome is 5,524. After excluding the four stop codons (UAA(*), UAG(*), AGA(*), AGG(*)), among the 64 codons, 31 codons have an RSCU value greater than 1, indicating that these codons are prioritized more highly. For instance, six codons (UUA(L), UUG(L), CUU(L), CUC(L), CUA(L), CUG(L)) coded for leucine with preference for UUA. RSCU values for these six codons were 1.68, 0.64, 1.47, 0.65, 0.99 and 0.56, respectively. The most commonly used codon is UUU-Phe (F), followed by UUA-Leu2 (L), AAA-Lys (K), and AUU-Ile (I). The least used amino acids are Ala (GCG) and Arg (CGU). Our results show that the codon distribution is largely consistent with the mitogenomes of Cobitinae studied previously (Yu et al. 2021).

**Table 2.** Codon usage in the mitochondrial genome of *Lepidocephalichthysberdmorei*.

Codon	Count	RSCU	Codon	Count	RSCU	Codon	Count	RSCU	Codon	Count	RSCU
UUU(F)	201	1.28	UCU(S)	103	1.37	UAU(Y)	118	1.19	UGU(C)	40	0.95
UUC(F)	113	0.72	UCC(S)	110	1.46	UAC(Y)	80	0.81	UGC(C)	44	1.05
UUA(L)	188	1.68	UCA(S)	68	0.9	UAA(*)	156	1.66	UGA(W)	75	1.18
UUG(L)	71	0.64	UCG(S)	40	0.53	UAG(*)	99	1.06	UGG(W)	52	0.82
CUU(L)	164	1.47	CCU(P)	110	1.01	CAU(H)	95	0.95	CGU(R)	21	0.65
CUC(L)	73	0.65	CCC(P)	134	1.23	CAC(H)	104	1.05	CGC(R)	37	1.15
CUA(L)	111	0.99	CCA(P)	137	1.26	CAA(Q)	156	1.34	CGA(R)	49	1.52
CUG(L)	63	0.56	CCG(P)	55	0.5	CAG(Q)	76	0.66	CGG(R)	22	0.68
AUU(I)	170	1.27	ACU(T)	102	1.06	AAU(N)	134	1.06	AGU(S)	49	0.65
AUC(I)	98	0.73	ACC(T)	120	1.25	AAC(N)	118	0.94	AGC(S)	82	1.09
AUA(M)	98	1.18	ACA(T)	128	1.33	AAA(K)	172	1.36	AGA(*)	75	0.8
AUG(M)	68	0.82	ACG(T)	35	0.36	AAG(K)	81	0.64	AGG(*)	45	0.48
GUU(V)	73	1.6	GCU(A)	82	1.13	GAU(D)	63	0.92	GGU(G)	44	0.81
GUC(V)	26	0.57	GCC(A)	114	1.57	GAC(D)	74	1.08	GGC(G)	53	0.98
GUA(V)	56	1.22	GCA(A)	75	1.03	GAA(E)	83	1.06	GGA(G)	70	1.3
GUG(V)	28	0.61	GCG(A)	20	0.27	GAG(E)	74	0.94	GGG(G)	49	0.91

### ﻿Transfer and ribosomal RNA genes

**Figure 3.**
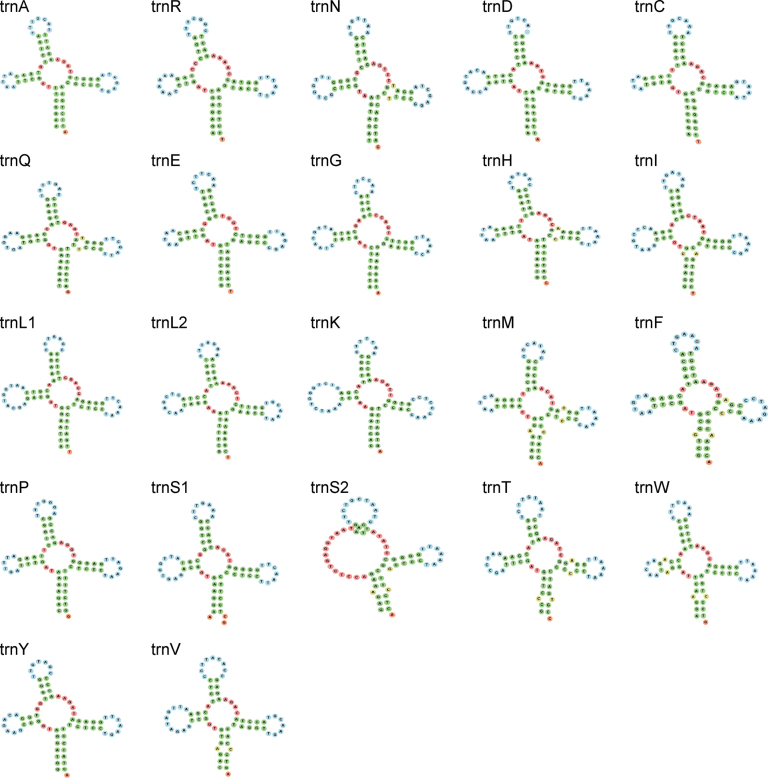
Putative secondary structure of tRNAs. Stems (typically helical) are shown in green, multiple loops (junctions) are shown in red, interior loops are shown in yellow, hairpin loops are shown in blue, and 5’ and 3’ unpaired regions are shown in orange.

The complete mitogenome of *L.berdmorei* contains 22 tRNA genes with a size of 1,559 bp, 14 of which are located on the H-strand while the others are on the L-strand (Table 1). The 22 tRNA genes range from 67 bp to 76 bp in length, of which the shortest was *tRNA^Cys^* (67 bp) and the longest was *tRNA^Lys^* (76 bp). The color in Fig. 3 represents the type of tRNA structure in which the nucleotide is located. All tRNA genes have a typical cloverleaf secondary structure except *tRNA^Ser^*^(*GCT*)^ lacking the Dihydrouracil (DHU) stem (Fig. 3). It is a common feature in many mitogenomes of metazoans, and can be integrated into ribosomes by adjusting its structure and function to fulfil its function of carrying and translocating amino acids (Watanabe et al. 2014; Liu et al. 2021; Xing et al. 2022).

The most prevalent non-Watson-Crick base pairs in the secondary structure of tRNAs are A-C (e.g., trnI, trnH, trnM, trnV, trnS1, trnT, trnW, and trnF), followed by T-T (trnQ and trnN), which are mostly located in the DHU, anticodon stems and acceptor (Fig. 3). And these mismatches may be modified by post-transcriptional editing processes without causing amino acid transport disorders (Tomita et al. 1996).

The lengths of *12S rRNA* and *16S rRNA* genes were 950 bp and 1,676 bp,which are located on the H strand (Table 1). They are bordered by *tRNA^Phe^* and *tRNA^Leu^*^(*TAA*)^ and separated by *tRNA^Val^*. Both the lengths and base compositions of *12S rRNA* and *16S rRNA* are almost identical among the reported Cobitidae fishes (Kottelat and Lim 1993; Nalbant 1993; Yu et al. 2016; Shen et al. 2020; Chu et al. 2022; Ke et al. 2023). It shows a positive AT-skew (0.23) and a negative GC-skew (-0.07) (Suppl. material 1: table S3). Compared to entire mitochondrial genome, the *16S rRNA* is a non-coding gene that evolves slowly, and it contains sufficient number of polymorphisms to distinguish species (Lakra et al. 2009; Sarri et al. 2014; Hossain et al. 2019). The *12S rRNA* is also frequently considered as a DNA meta barcoding in fish identification and phylogenetic studies (Miya et al. 2015).

### ﻿Control region

The only large control region of *L.berdmorei* mitogenome is the D-loop, located between the *tRNA^Pr^*^o^ and *tRNA^Phe^*, with a length of 922 bp (Fig. 1, Table 1). It plays a role in the regulation of replication and transcription and is the most rapidly evolving and changing region of the mitochondrial genome (Clayton 1982, 1991; Shadel and Clayton 1997; Zhou et al. 2014; Gao et al. 2023). The A+T-rich content of the *L.berdmorei* D-loop region is 66.27%, which is higher than the average value of the whole mitogenome (58.43%) and 13 PCGs (56.11–61.07%) (Suppl. material 1: table S3), as found in other vertebrates (Brown et al. 1986; Saccone et al. 1987; Zou et al. 2017; Ke et al. 2023).

In addition to gene duplication and insertion/deletion events, the main cause of mitochondrial genome size variation is differences in control region length (Mignotte et al. 1990; Lee et al. 1995; Pereira 2000; Minhas et al. 2023). Previous studies have demonstrated that tandem repeat sequences are prevalent in the D-loop of teleost lineage (Lee et al. 1995; Nicholls and Minczuk 2014; Jemt et al. 2015; Xu et al. 2016; Ke et al. 2023). It is worth noting that the copy number not only varies between species, but also among individuals within the same species (Norman et al. 1994; Lunt et al. 1998; Boore 1999; Xu et al. 2021). Thus, compared with the complex and large eukaryotic genome, the mitochondrial genome is simple in structure with shorter sequences, contains both conserved and highly variable regions, and can be used for taxonomic identification of species at different levels of evolution (Pereira et al. 2008; Jamandre et al. 2014; Nicholls and Minczuk 2014; Jemt et al. 2015; D’Souza and Minczuk 2018). Nevertheless, multiple duplicate regions have been found in some species that may adversely affect PCR amplification, sequencing, or both (Singh et al. 2008; Cadahía et al. 2009). As a result, researchers have avoided using this region for phylogenetic purposes, focusing instead on *rRNA* or PCGs (Slechtová et al. 2008; Wang et al. 2021; Sureandiran et al. 2023; Zhang et al. 2023).

### ﻿Phylogenetic analysis

**Figure 4.**
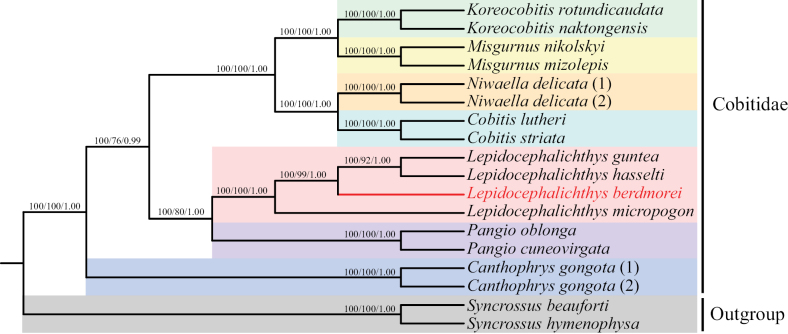
Phylogenetic tree of Cobitidae and two outgroups based on the NJ, ML and BI analysis of 13 concatenated protein-coding genes. Tree topologies produced by NJ, ML methods, and BI analysis were equivalent. The numbers at the nodes represent bootstrap support values for NJ and ML analyses and Bayesian posterior probability, sequentially, and the red branch represents the specie in this study.

Cobitidae belongs to Osteichthyes, Cypriniformes, and has three subfamilies: Nemacheilinae, Botiinae and Cobitinae (Hora 1932; Nalbant 1993; Tang et al. 2005; Slechtová et al. 2008; Chu et al. 2022). Sawada (1982) proposed a phylogeny of the Cobitoidea (limited to loaches) as (Botiinae + Cobitinae) + (Nemacheilinae + Homalopterinae) based on 52 osteological characters. Nevertheless, due to their morphological similarity and frequent overlap, differentiating species within Cobitidae based solely on morphology is a challenging endeavor (Kottelat and Lim 1993; Nalbant 1993; Shen et al. 2020; Ke et al. 2023). In order to determine the phylogenetic status of *L.berdmorei* in the family Cobitidae, 17 complete mitochondrial genomes from the GenBank database were selected to reconstruct phylogenetic trees. Based on the 13 PSGs concatenated dataset, the NJ, ML and BI phylogenies generated identical topology with high bootstrap support and posterior probability values, respectively (Fig. 4). All trees presented two major clades corresponding to the outgroup. *Canthophrys* is located at the base of the phylogenetic tree. Our results are generally consistent with the traditional morphological classification and recent molecular studies (Hora 1932; Slechtová et al. 2008; Sudasinghe et al. 2024).

Firstly, the phylogenetic tree revealed that *L.guntea*, *L.hasselti*, and *L.berdmorei* clustered as a monophyletic clade, followed by a clade with *L.micropogon* with high bootstrap support. Secondly, the genus *Lepidocephalichthys* and *Pangio* which formed a sister branch with high bootstrap support and posterior probability values, which was consistent with the previous study (Slechtová et al. 2008; Yu et al. 2021). Notably, Slechtová et al. (2008) found that the genera *Lepidocephalichthys* and *Pangio* were considered as a sister group in the RAG-1 phylogeny; but this relationship was not supported by the *cytb* dataset. Meanwhile, based on *cyt b* and RAG-1 datasets, these four genera of Cobitidae (*Cobitis*, *Niwaella*, *Misgurnus*, and *Koreocobitis*) form a distinct monophyletic group (Slechtová et al. 2008). Generally, from the phylogenetic tree of genetic evolution, the evolutionary status of *L.berdmorei* was defined.

## ﻿Conclusions

In conclusion, the complete mitochondrial DNA sequence of *L.berdmorei* is determined for the first time by the primer walking sequence method. The mitogenome is 16,574 bp in length, and encodes all of the 37 genes that are typical for Cobitidae fish. We compared mtDNA from *L.berdmorei* with that of other teleost and analyzed mitogenome composition, PCGs, and codon usage, transfer and ribosomal RNA genes, and noncoding regions (control region, intergenic spacers). The generated phylogenetic trees yielded convincing evidence that the genus *Lepidocephalichthys* formed a monophyletic group. These findings will provide new insights into better understanding the phylogenetic status of this intriguing and ecologically important group.

## ﻿Additional information

### Author ORCIDs

Ying Wang https://orcid.org/0000-0002-8222-7510

### Data availability

Genome sequence data that support the findings of this study are openly available from the GenBank at https://www.ncbi.nlm.nih.gov/, under accession No. OP651767.

## Supplementary materials

Supplementary material 1 Supplementary files Data type docx Explanation note **figure S1.** Images of biological sample for this study. **table S1.** Primers used for amplification of the mitochondrial genome of *Lepidocephalichthysberdmorei*. **table S2.** Species and GenBank accession numbers of mitogenomes used in this study. **table S3.** Nucleotide contents of genes and the mitochondrial genome skew of *Lepidocephalichthysberdmorei*. https://binary.pensoft.net/file/1195360This dataset is made available under the Open Database License (http://opendatacommons.org/licenses/odbl/1.0/). The Open Database License (ODbL) is a license agreement intended to allow users to freely share, modify, and use this Dataset while maintaining this same freedom for others, provided that the original source and author(s) are credited.Min Zhou, Cheng Wang, Ziyue Xu, Zhicun Peng, Yang He, Ying Wang
