# Supplementary material for: ﻿Complete mitochondrial genome of Lepidocephalichthysberdmorei and its phylogenetic status within the family Cobitidae (Cypriniformes)
Source: Zookeys. 2024 Dec 10;1221:51–69. doi: 10.3897/zookeys.1221.129136 (PMC11653074; doi:10.3897/zookeys.1221.129136)
Supplement: Supplementary material 1 — Supplementary files [file zookeys-1221-051_article-129136__-s001.docx]

Figure S1. Images of biological sample for this study.


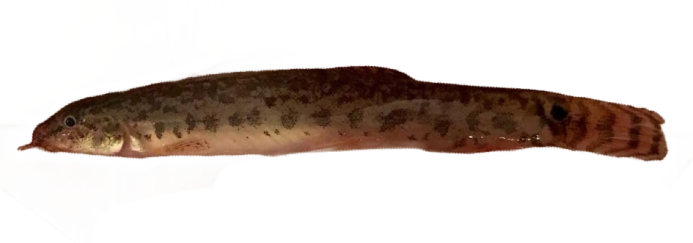


Table S1. Primers used for amplification of the mitochondrial genome of

*Lepidocephalichthys berdmorei.*

| Fragment | Gene | Primer | Sequence (5’-3’) | Length (bp) |
| --- | --- | --- | --- | --- |
| F1 | *12S rRNA*-*16S rRNA* | SLF1 | GACGAGGAGCAGGCATCAGG | 2,473 |
|  |  | SLR1 | GTTACAGATAGAAACTGACC |  |
| F2 | *16S rRNA*-*tRNA^Asn^* | SLF2 | GGTTTACGACCTCGATGTTG | 2,751 |
|  |  | SLR2 | CTAAGAGTTTGTAGGATCGAG |  |
| F3 | *tRNA^Trp^*-*cox2* | SLF3 | CTTCAAAGCTCTAAGCAGGAG | 2,762 |
|  |  | SLR3 | CCTAGTGAGGCGTCTTCTAG |  |
| F4 | *cox2*-*nd3* | SLF4 | GTTAAGCTAGATGGTGTTCC | 2,025 |
|  |  | SLR4 | GATACAATTGCTAGGATAAGTG |  |
| F5 | *cox3*-*tRNA^Leu(TAG)^* | SLF5 | CTCTATCTACTGATGAGGTTC | 2,391 |
|  |  | SLR5 | CTTGGATTTGCACCAAGAGT |  |
| F6 | *tRNA^Leu(TAG)^*-*nd5* | SLF6 | GGATAACAGCTCATCCATTG | 1,273 |
|  |  | SLR6 | GTCTCAATGATGGCGTCTTTCG |  |
| F7 | *nd5*-D-loop | SLF7 | ATGACGAACAAGATATCCGA | 2,736 |
|  |  | SLR7 | GTTTGCAAGAATTGATAGGG |  |
| F8 | *tRNA^Thr^*-*12S rRNA* | SLF8 | GTAATCCGAAGATCGGAGGT | 1,356 |
|  |  | SLR8 | CGTATAACCGCGGTGGCTGG |  |

Table S2. Species and GenBank accession numbers of mitogenomes used in this study.

| Scientific name | Accession ID | Sequence length (bp) | References |
| --- | --- | --- | --- |
| *Lepidocephalichthys berdmorei* | OP651767 | 16,574 | This study |
| *Lepidocephalichthys guntea* | AP011338 | 16,567 | Unpublished |
| *Lepidocephalichthys hasselti* | AP013334 | 15,897 | Unpublished |
| *Lepidocephalichthys micropogon* | NC_031595 | 16,608 | Unpublished |
| *Koreocobitis rotundicaudata* | AP011339 | 16,569 | Unpublished |
| *Koreocobitis naktongensis* | HM535625 | 16,567 | Unpublished |
| *Misgurnus nikolskyi* | AB242171 | 16,570 | Saitoh et al. 2006 |
| *Misgurnus mizolepis* | MF579258 | 16,571 | Unpublished |
| *Niwaella delicata* (1) | AP009308 | 16,571 | Saitoh et al. 2010 |
| *Niwaella delicata* (2) | AP011230 | 16,572 | Unpublished |
| *Cobitis lutheri* | NC_022717 | 16,639 | Unpublished |
| *Cobitis striata* | AP010782 | 16,646 | Saitoh et al. 2010 |
| *Pangio oblonga* | NC_031592 | 16,600 | Unpublished |
| *Pangio cuneovirgata* | NC_031594 | 16,596 | Unpublished |
| *Canthophrys gongota* (1) | AP011289 | 16,561 | Unpublished |
| *Canthophrys gongota* (2) | NC_031576 | 16,561 | Unpublished |
| *Syncrossus beauforti* | NC_031546 | 16,567 | Unpublished |
| *Syncrossus hymenophysa* | NC_033951 | 16,513 | Unpublished |

Table S3. Nucleotide contents of genes and the mitochondrial genome skew of *Lepidocephalichthys berdmorei*.

| Regions | Size  (bp) | A(%) | T(%) | C(%) | G(%) | A+T(%) | G+C(%) | AT-skew | GC-skew |
| --- | --- | --- | --- | --- | --- | --- | --- | --- | --- |
| rRNAs | 2,626 | 34.20 | 21.29 | 23.80 | 20.72 | 55.48 | 44.52 | 0.23 | -0.07 |
| *nd1* | 975 | 28.10 | 31.79 | 26.36 | 13.74 | 59.90 | 40.10 | -0.06 | -0.31 |
| tRNAs | 1,559 | 30.53 | 24.70 | 25.59 | 19.18 | 55.23 | 44.77 | 0.11 | -0.14 |
| *nd2* | 1,047 | 31.33 | 27.60 | 28.65 | 12.42 | 58.93 | 41.07 | 0.06 | -0.40 |
| *cox1* | 1,551 | 25.85 | 31.98 | 24.24 | 17.92 | 57.83 | 42.17 | -0.11 | -0.15 |
| *cox2* | 691 | 27.93 | 29.38 | 25.33 | 17.37 | 57.31 | 42.69 | -0.03 | -0.19 |
| *atp8* | 168 | 33.93 | 26.79 | 26.79 | 12.50 | 60.71 | 39.29 | 0.12 | -0.36 |
| *atp6* | 684 | 27.63 | 32.16 | 25.73 | 14.47 | 59.80 | 40.20 | -0.08 | -0.28 |
| *cox3* | 786 | 26.08 | 30.03 | 27.35 | 16.54 | 56.11 | 43.89 | -0.07 | -0.25 |
| *nd3* | 351 | 25.93 | 31.91 | 25.93 | 16.24 | 57.83 | 42.17 | -0.10 | -0.23 |
| *nd4l* | 297 | 24.92 | 33.00 | 25.93 | 16.16 | 57.91 | 42.09 | -0.14 | -0.23 |
| *nd4* | 1,383 | 27.91 | 30.15 | 27.04 | 14.90 | 58.06 | 41.94 | -0.04 | -0.29 |
| *nd5* | 1,839 | 29.96 | 31.10 | 25.56 | 13.38 | 61.07 | 38.93 | -0.02 | -0.31 |
| *nd6* | 522 | 41.76 | 16.67 | 30.46 | 11.11 | 58.43 | 41.57 | 0.43 | -0.47 |
| *cytb* | 1,141 | 27.52 | 33.04 | 25.07 | 14.37 | 60.56 | 39.44 | -0.09 | -0.27 |
| D-loop | 922 | 32.32 | 33.95 | 19.74 | 13.99 | 66.27 | 33.73 | -0.02 | -0.17 |
| PCGs | 11,413 | 28.65 | 30.27 | 26.29 | 14.79 | 58.92 | 41.08 | -0.03 | -0.28 |
| Mitogenome | 16,574 | 29.90 | 28.54 | 25.46 | 16.10 | 58.43 | 41.57 | 0.02 | -0.23 |
